# Case Report: Primary Diffuse Leptomeningeal Oligodendrogliomatosis in a Young Adult Cat

**DOI:** 10.3389/fvets.2021.795126

**Published:** 2021-12-15

**Authors:** Elisa Chludzinski, Christina Puff, Jürgen Weber, Marion Hewicker-Trautwein

**Affiliations:** ^1^Department of Pathology, University of Veterinary Medicine, Hannover, Germany; ^2^Tierärztliche Praxis für Kleintiere Dr. med. vet. Jürgen Weber, Oer-Erkenschwick, Germany

**Keywords:** CNS, feline, glioneuronal tumor, meninges, OLIG2, oligodendroglioma, PDLG

## Abstract

A 2-year-old cat was presented with progressive ataxia. Despite treatment the animal died. Pathomorphological examination revealed a widespread leptomeningeal mass at all levels of the central nervous system accentuated on the cervical spinal cord and the medulla oblongata without presence of a primary intraaxial tumor. The neoplasm was mainly composed of round, uninucleate cells with hyperchromatic nuclei, which were immunopositive for OLIG2, doublecortin, MAP2, synaptophysin, and vimentin, indicating components of both oligodendroglial and neuronal differentiation. Ki-67 immunohistochemistry indicated a high proliferation activity of the neoplasm. Few GFAP positive and Iba-1 positive cells were interpreted as reactive astrocytes and macrophages or microglia, respectively. The tumor was immunonegative for CD3, CD20, PAX5, MUM1, pan-cytokeratin, S100, NSE, p75^NTR^, NeuN and periaxin. These findings led to the diagnosis of primary diffuse leptomeningeal oligodendrogliomatosis. This is the first reported case of this entity in a young cat, which should be considered as a differential diagnosis for diffuse subarachnoidal round cell infiltrates.

## Introduction

Gliomas are the most common primary intracranial neoplasms in humans and, after meningiomas, the second most frequent primary CNS neoplasm in dogs (1, 2). They are uncommon to rare in cats (3–7).

Based on their morphological appearance and immunohistochemical profile, gliomas have been classically grouped into astrocytomas, oligodendrogliomas, and ependymomas with various subtypes. During recent years, genetic alterations have gained importance in classification of human gliomas, which have not been determined yet in animals (8).

Commonly, glial neoplasms develop as intraaxial tumors within the telencephalic white and gray matter including basal nuclei, the diencephalon (thalamus, hypothalamus) and the mesencephalon with only rare occurrence in the pons, medulla oblongata and the spinal cord. Especially anaplastic variants and glioblastomas (WHO grade III and IV) may secondarily infiltrate into adjacent structures, including the leptomeninges. Rarely, this is followed by further diffuse spread via the cerebrospinal fluid (CSF), resulting in secondary diffuse leptomeningeal gliomatosis (9–14).

In contrast, primary diffuse leptomeningeal gliomatosis (PDLG) is an even rarer manifestation which is characterized by a diffuse infiltration of the subarachnoid space by neoplastic glial cells without evidence of a primary intraaxial tumor (15).

PDLG was firstly described in humans in 1923 and in dogs in 2013 (16–18). Recently, a case of primary leptomeningeal gliomatosis was described in an older cat (19).

Due to its rarity and unspecific clinical and radiological findings, an intravital diagnosis, predominantly based on MRI findings and exclusion of other diseases, is challenging and requires histopathological confirmation (15, 20, 21). In human medicine, adequate meningeal biopsy is a useful tool allowing a definitive diagnosis (22). However, due to the aggressive nature of the tumor, short clinical course and poor prognosis, in most cases final diagnosis of PDLG is made at necropsy (20).

The present report describes the clinical, pathomorphological, and immunohistochemical findings in a young cat with primary diffuse leptomeningeal oligodendrogliomatosis.

## Case Description

### Clinical History

A 2-year-old, female-neutered domestic shorthair cat of 3.43 kg body weight was presented with acute back pain and mild ataxia after a jump from a cupboard. Clinical examination revealed no additional specific findings. A presumptive diagnosis of suspect traumatic injury of vertebral bone and/or spinal cord was made and the cat was treated with robenacoxib (Onsior™ 1.75 mg/kg, s.i.d.). After 2 days without clinical improvement, blood count and blood chemistry were performed and revealed a mild hypophosphatemia (3.1 mg/dl, reference range: 3.4–8.5 mg/dl) and monocytosis (6.6%, reference range: 1–3%). A radiological examination of the thorax, abdomen and the proximal hind limbs showed a mildly increased interstitial pattern in the craniodorsal lung, but no abnormalities in the skeleton or the spinal cord (Supplementary Figure 1A). The cat was further treated with meloxicam (Metacam™ 0.05 mg/kg s.i.d.), amoxicillin (Duphamox LA™ 15 mg/kg once every other day) and vitamin B (B-Vitamin-Tabletten™, one pill s.i.d.). Four days after initial presentation, the cat showed mild apathy and severe ataxia with markedly reduced proprioceptive reactions in all four limbs and reduced segmental reflexes in the thoracic limbs. The suspected neuroanatomical localization was the brain or the spinal cord cranial to T2. No cells were found in the cerebrospinal fluid (CSF). An infection with feline coronavirus, bornavirus, *Toxoplasma gondii* and *Bartonella henselae* within the CSF was excluded via PCR. Computed tomography (CT) of the entire body was performed but showed no abnormalities of the central nervous system (Supplementary Figures 1B,C). Therefore, the cause of neurological deficits remained undefined. The cat was hospitalized and treated with Ringer infusion (Deltamedica™ 5 ml/kg body weight during the entire time of hospitalization). Further progressive clinical decline with irresponsiveness to treatment resulted in lateral recumbency and death, which was 6 days after initial presentation. The cat was subsequently submitted to the Department of Pathology for pathological examination (necropsy, histopathological examination, immunohistochemistry).

### Pathomorphological Findings

**Figure 1:**
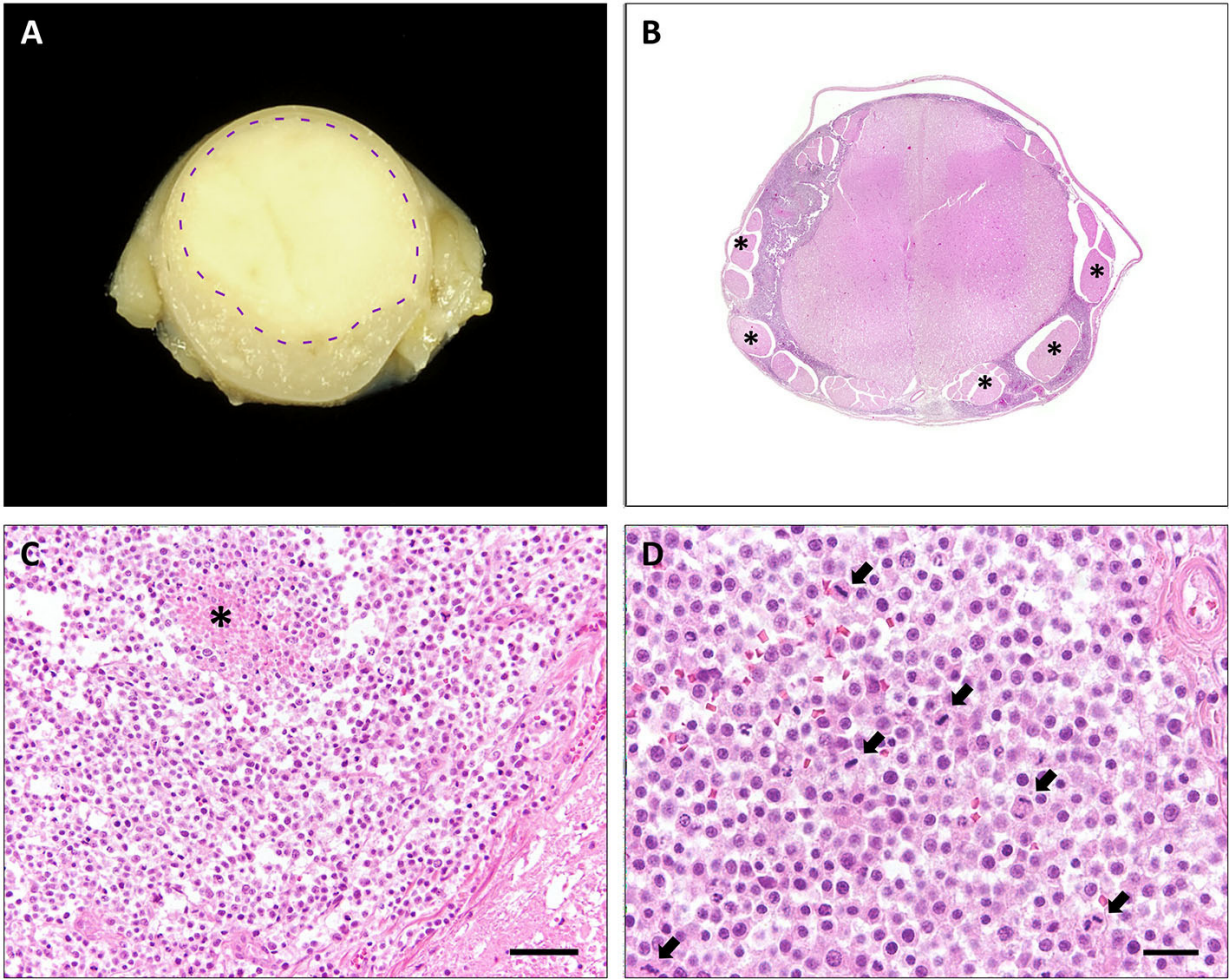
Cervical spinal cord, cat. **(A)** Transversal section of formalin-fixed spinal cord with leptomeningeal pale, gray-beige mass (dashed line: junction tumor-spinal medulla). **(B)** Diffuse, basophilic cell infiltrates and edema within leptomeninges with compression of spinal cord. Spinal nerves (asterisks) are omitted by the tumor. HE stain. **(C)** Neoplastic infiltrates with junction to spinal medulla (bottom right) and necrosis (asterisk), HE stain. Scale bar: 50 μm. **(D)** High mitotic activity within the neoplasm (arrows), HE stain. Scale bar: 20 μm.

At gross examination, a diffuse enlargement of the leptomeninges was noted, mostly pronounced within cervical segments with rostral extension to the medulla oblongata. After formalin fixation, cross sections revealed a well demarcated, gray-beige, soft, subdural thickening within the whole circumference of the spinal cord, involving primarily the cervical part, and the medulla oblongata with partial compression of the neuroparenchyma (Figure 1A). Macroscopically, no intraaxial tumor was found in coronal sections of the brain and spinal cord.

All other organs lacked significant macroscopic findings.

Histopathological examination of representative localizations of the CNS (cerebral cortex, hippocampus, cerebellum, mesencephalon, medulla oblongata, and cervical, thoracic, lumbar, and sacral spinal cord) revealed a restriction of the mass to the subarachnoidal space (Figure 1B). It was composed of closely packed, round, uninucleated cells arranged in sheets, accompanied by low amounts of fine fibrovascular stroma. The medium-sized cells possessed variably distinct cell borders and contained low amounts of finely granular eosinophilic cytoplasm. Nuclei measured 10–15 μm in diameter, were centrally to eccentrically located, round to oval and frequently hyperchromatic with one distinct small nucleolus. Multifocally, large necrotic areas were present (Figure 1C). There was mild anisocytosis and –karyosis with a mitotic count of 20 mitoses per 2.37 mm^2^ (Figure 1D). In two localizations, the tumor infiltrated superficially the adjacent neuroparenchyma of the dorsal medulla oblongata and the cerebral cortex.

The neuroparenchyma adjacent to the tumor multifocally showed compression and marked degenerative changes including spheroid formation and vacuolation of the white and gray matter.

### Immunohistochemical Findings

**Figure 2:**
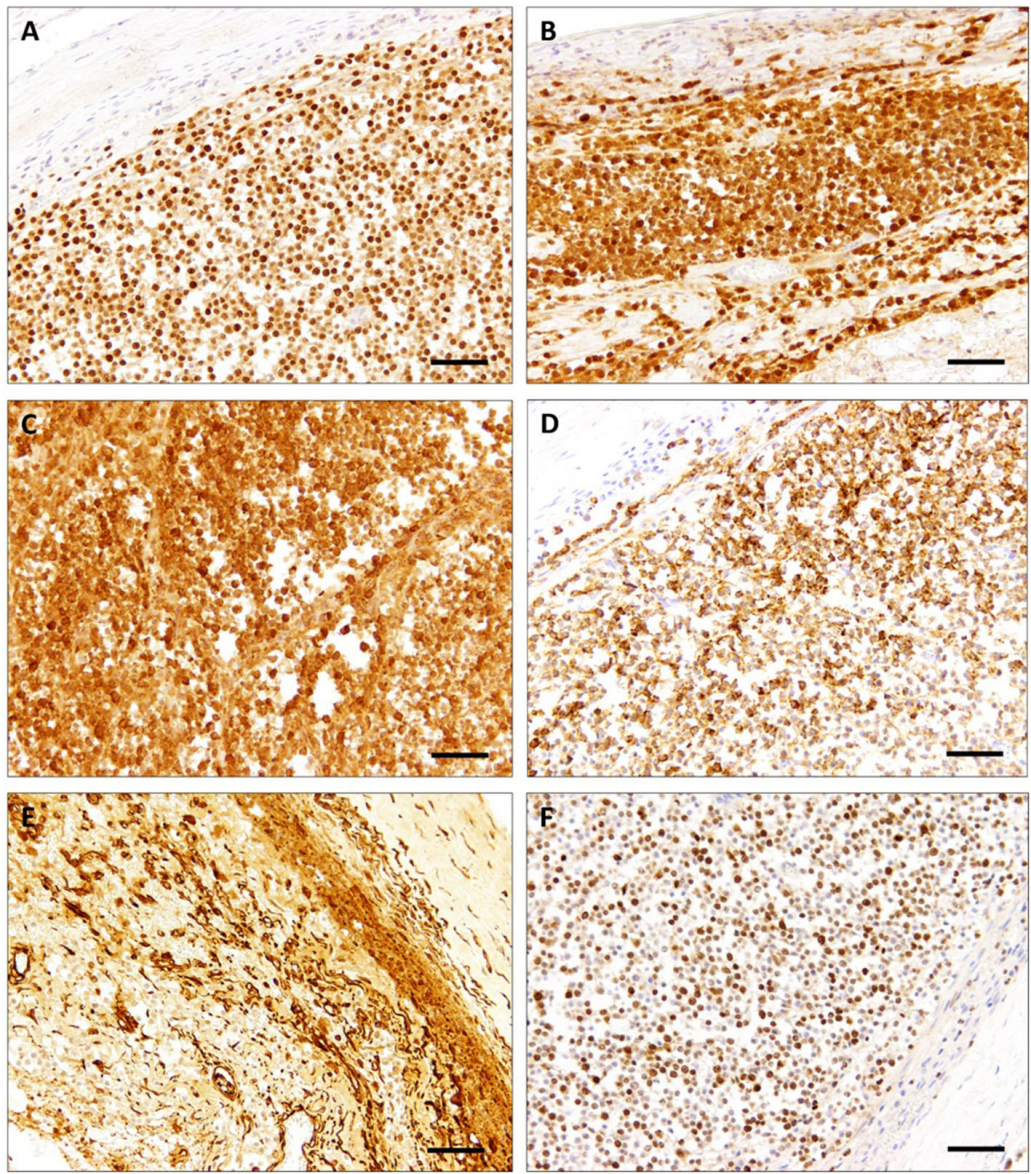
Spinal cord, cat. Tumor cells are diffusely immunopositive for OLIG2 **(A)**, doublecortin **(B)**, and MAP2 **(C)**. The majority of tumor cells stained positive with synaptophysin **(D)**. Positive vimentin reaction is mainly restricted to tumor—associated vasculature **(E)**. High proliferation activity was demonstrated with positive nuclear staining for Ki-67 **(F)**. Scale bars: 50 μm.

In order to phenotype the tumor cells, selected representative paraffin-embedded samples of the brain and the spinal cord were subjected to immunohistochemistry. The respective primary antibodies and information referring to antigen-retrieval are listed in Table 1. Neoplastic cells showed a diffuse, intranuclear expression of oligodendrocyte transcription factor 2 (OLIG2) and doublecortin (Figures 2A,B). Moreover, tumor cells exhibited a diffuse cytoplasmic expression of microtubule-associated protein 2 (MAP2) and synaptophysin (Figures 2C,D). Some of the neoplastic cells showed a cytoplasmic vimentin expression (Figure 2E). Ki-67 as a proliferation marker protein was detected in more than 50% of the neoplastic cells (Figure 2F). Scattered ionized calcium-binding adapter molecule 1 (Iba1)-positive cells and few scattered glial fibrillary acid protein (GFAP)-positive cells were present within the neoplasm. Neoplastic cells lacked immunoreactivity for neurofilament (NF), S100 protein, neuron specific enolase (NSE), p75 neurotrophin receptor, neuronal nuclear protein (NeuN), periaxin, pan-cytokeratin, CD3, CD20, CD79a, paired box 5 transcription factor (PAX5), and multiple myeloma oncogene 1 (MUM1).

**Table 1:** Antibodies used for immunohistochemistry and their reactivity within the tumor.

**Epitope**	**Source**	**Cat No**	**Species**	**Clone**	**Dilution**	**Pretreatment**	**Cell reaction**
OLIG2	Abcam, Cambridge, UK	ab109186	Rabbit	EPR2673	1:2000	HIER	+ + +, nuclear
Doublecortin	Santa Cruz Biotechnology, Dallas, TX, USA	sc-271390	Mouse	E-6	1:100	HIER	+ + +, cytoplasmic
MAP2	Sigma-Aldrich, Taufkirchen, Germany	M1406	Mouse	AP-20	1:800	HIER	+ + +, cytoplasmic
Synaptophysin	Dako, Hamburg, Germany	M7315	Mouse	DAK-SYNAP	1:500	HIER	+ +, cytoplasmic
Vimentin	Dako, Hamburg, Germany	M0725	Mouse	V9	1:100	–	+, cytoplasmic
Ki-67	Dako, Hamburg, Germany	M7240	Mouse	MIB-1	1:100	HIER	>50%, nuclear
GFAP	Dako, Hamburg, Germany	Z0334	Rabbit	Polyclonal	1:1000	–	Scattered, cytoplasmic
Iba-1	Invitrogen, Thermo Fisher Scientific, Langenselbold, Germany	PA5-27436	Rabbit	Polyclonal	1:1000	HIER	scattered, cytoplasmic
NF	Biolegend, San Diego, CA, USA	837904	Mouse	SMI 312	1:4000	–	Negative
NSE	Dako, Hamburg, Germany	M0873	Mouse	BBS/NC/VI-H14	1:100	–	Negative
NeuN	Millipore, Burlington, MA, USA	MAB377	Mouse	A60	1:1600	HIER	Negative
S100	Sigma-Aldrich, Taufkirchen, Germany	S2644	Rabbit	Polyclonal	1:800	–	Negative
Periaxin	Sigma-Aldrich, Taufkirchen, Germany	A06390	Rabbit	Polyclonal	1:2000	HIER	Negative
p75^NTR^	American Tissue Culture Collection, Rockville, MD, USA	200-3-G6-4 (20.4)	Mouse	HB8737	1:5	–	Negative
Pan-CK	Dako, Hamburg, Germany	M3515	Mouse	AE1/AE3	1:500	HIER	Negative
CD3	Dako, Hamburg, Germany	A0452	Rabbit	Polyclonal	1:200	HIER	Negative
CD20	Lab Vision, Thermo Fisher Scientific, Langenselbold, Germany	RB-9013-P1	Rabbit	Polyclonal	1:300	HIER	Negative
CD79a	Abcam, Cambridge, UK	ab62650	Mouse	HM57	1:5000	HIER	Negative
PAX5	Biolegend, San Diego, CA, USA	649702	Rat	1H9	1:500	HIER	Negative
MUM-1	Dako, Hamburg, Germany	M7259	Mouse	MUM1p	1:10	HIER	Negative

*OLIG2, oligodendrocyte transcription factor 2; MAP2, microtubule-associated protein 2; GFAP, glial fibrillary acid protein; Iba-1, ionized calcium-binding adapter molecule 1; NF, neurofilament; NSE, neuron-specific enolase; p75^NTR^, p75 neurotrophin receptor; Pan-CK, pan-cytokeratin; CD, cluster of differentiation; PAX5, paired box 5 transcription factor; MUM-1, multiple myeloma oncogene 1; Cat No, catalog number; HIER, heat-induced epitope retrieval with citrate buffer (pH 6.0) and microwave (800W for 20 min); immunopositivity of tumor cells: + + +, >80%; ++, >50%; +, <50%*.

## Discussion

The morphological findings in the present case, in particular the absence of a primary intraaxial tumor and the immunostaining profile, are consistent with primary diffuse leptomeningeal oligodendrogliomatosis, which is a tumor that is mainly composed of oligodendroglial neoplastic cells.

In general, tumors of oligodendroglial origin are histopathologically characterized by uniform, densely packed cells with vacuolated or eosinophilic cytoplasm, a round hyperchromatic nucleus and distinct cell borders with variable patterns of cell arrangement. Not infrequently, delayed formalin fixation causes a perinuclear halo, resulting in a “honeycomb pattern” appearance (23). However, tumor cell morphology and origin are variable and the tumor might resemble other tumor types, including neuronal tumors and lymphomas, which have to be excluded via immunohistochemistry.

The present tumor was diffusely immunopositive for OLIG2, doublecortin, MAP2, synaptophysin, vimentin, and Ki-67 (24). Albeit being expressed in other gliomas, the proportion of OLIG2 positive cells in oligodendroglial tumors in humans and dogs is significantly higher compared to astrocytomas and oligoastrocytomas (24, 25). Positive reaction with OLIG2 has been demonstrated in oligodendrogliomatosis of other species and the first reported feline case of gliomatosis (16, 17, 19, 26, 27).

Doublecortin is expressed in invasive brain tumors in humans, in canine oligodendroglioma, anaplastic meningiomas, and leptomeningeal oligodendrogliomatosis (16, 28–30), which might give support to the theory that neuronal progenitors might contribute to the development of these tumors.

Microtubule-associated protein 2 (MAP2) immunopositivity was reported in canine oligodendroglioma, (31) in the previously described case of a cat with primary leptomeningeal gliomatosis, and an African hedgehog with an oligodendroglioma with neuronal differentiation (19, 31, 32).

Synaptophysin immunopositivity, as observed in the present case, has been reported in human and canine oligodendrogliomas and diffuse leptomeningeal glioneuronal tumors (8, 10, 33) as well as in an oligendroglioma with neuronal differentiation in an African hedgehog (32) and two dogs (34). However, immunopositivity seems to be variable, as there are also reports of oligodendroglial tumors and primary diffuse leptomeningeal oligodendroglial tumors which were immunonegative for synaptophysin (35, 36).

Vimentin expression in oligodendrogliomas is variable, but has been reported in the recently published feline case of primary leptomeningeal gliomatosis, a canine brain oligodendroglioma, a canine leptomeningeal spinal oligodendroglioma, and in human cases of PDLG (15, 16, 19, 20, 37). It was negative in a bovine and canine case of diffuse leptomeningeal oligodendrogliomatosis (26, 27). Predominantly, it stains vascular elements of the tumor stroma.

The present tumor showed a high labeling index for the proliferation marker Ki-67. Although it is often highly expressed in anaplastic glial tumors of cats, Ki-67 expression in feline gliomas does not always correlate with the grade of the tumor (12, 38). In the recently published case of feline primary diffuse leptomeningeal gliomatosis, approximately 5% of tumor cells were positive for Ki-67 (19).

The scattered GFAP- and Iba1-positive cells in our case were interpreted as reactive astrocytes and macrophages/microglia, respectively.

The present tumor was immunonegative for NF, NSE, NeuN, and S100. These markers are variably expressed in oligodendroglial tumors of domestic animals and humans (16, 26, 27, 33, 36, 37, 39, 40).

Immunonegativity of the present tumor for periaxin and p75^NTR^, pan-cytokeratin, and CD3, CD20, CD79a, PAX5, and MUM1 excluded nerval, epithelial, and lymphocytic origin, respectively.

At large, the variable and inconsistent immunohistochemical findings regarding neuronal markers in oligodendrogliomas and PDLG imply that oligodendrogliomas and oligodendroglioma-like tumors might arise from common glioneuronal progenitor cells (41, 42). This theory is supported by the fact that classic oligodendrogliomas may present with neurocytic rosettes and neurocytoma, a rare intracranial neuroepithelial tumor in humans, can show 1p/19q deletion, a mutation commonly found in human oligodendroglia-like tumors (33, 43).

It has been postulated that PDLG originates from heterotopic glial nests, which are small aggregates of glial cells within the subarachnoid space arising from protrusions of mature glia cells from the neuraxis. They are most frequently found at the level of the medulla oblongata and the lumbar spinal cord, in approximately 1% of random necropsies of humans. The pronounced manifestation of the tumor within the subarachnoidea of the medulla oblongata in the present case lends support to this theory. However, although glial heterotopias were detected in a human case of primary diffuse leptomeningeal oligodendroglioma at the brain base, these tumors can also manifest in other localizations along the entire CNS (20, 44).

In the current human WHO classification of tumors of the nervous system, various oligodendrogliomatous diffuse leptomeningeal masses either with or without primary intraaxial involvement and neuronal components, are categorized by specific genetic abnormalities and have recently been summarized under the term “primary diffuse glioneuronal tumor,” as the nosological position of these tumors remains controversial (8, 45).

In humans, there is an age predilection of diffuse leptomeningeal oligodendroglioma-like neoplasms toward children and young adults, although all ages are susceptible (45). Low-grade diffuse gliomas and oligodendrogliomas as well as diffuse leptomeningeal neuroepithelial tumors in children have been attributed to 1p and/or 19q loss with associated BRAF gain or mutation, resulting in an activated MAPK signaling pathway (46–48). In oligodendrogliomas, IDH1 or 2 mutations frequently occur (49, 50).

Regarding domestic animals, brachycephalic dog breeds are predisposed to develop oligodendroglioma with a suspected defect on chromosome 26 (2, 51). The exclusive representation of brachycephalic dogs (4 boxer dogs, 1 Staffordshire bull terrier, 1 Cane Corso) in published cases of canine diffuse leptomeningeal gliomatosis may suggest a similar breed disposition to PDLG (16, 17, 27, 52). However, further case data are required to confirm this assumption. For PDLG in domestic animals, no specific genetic alterations have been determined so far.

Clinical findings in humans and animals with leptomeningeal gliomatosis are relatively unspecific and are the sequel of impaired liquor circulation and compression of adjacent intraaxial structures (20, 39). In dogs and cats, progressive ataxia, decreased proprioception in all four limbs, tetraparesis and seizures are reported (16, 17, 19, 27, 52). CSF fluid in humans revealed elevated protein levels with low to moderate pleiocytosis and normal or low glucose levels (15, 53, 54), but neoplastic cells are not found in every CSF sample, probably due to their adhesion through cell processes, which makes them less prone to exfoliation (20, 54). This might also account for the present case, where CSF was lacking neoplastic cells. Neuroimaging techniques are a useful tool for intravital detection of CNS tumors with MRI being usually more sensitive than CT. The lower sensitivity of CT, which was used in the present case, compared to MRI might explain that the present tumor was not detected intravitally. Typical MRI findings in humans and animals in PDLG are diffuse leptomeningeal enhancement with no discernible intraaxial component (15, 17, 52). In a canine case of primary diffuse leptomeningeal oligodendrogliomatosis, a dural tail sign, which can also be found in various other masses, was detected in postcontrast T1W images (27). In addition, ventricular enlargement due to hydrocephalus and meningeal calcification might be observed (55, 56).

Histopathological examination of biopsy specimens is the only way to confirm a definite diagnosis *intra vitam* combined with MRI, however, repeated biopsies might be necessary to get a representative sample (15, 22, 57). PET-CT and intraoperative biopsy analysis can improve the effectivity of a representative sample in humans (58). Yet, to our knowledge, there are no reports of intravital histopathologic diagnosis of PDLG in domestic animals, which might be due to the clinical severity usually resulting in euthanasia.

Differentials in humans and domestic animals include other neoplasms, like secondary meningeal gliomatosis, ependymoma, pilocytic astrocytoma or multicentric neoplasia and meningitis of autoimmune or infectious etiology (15, 17, 20, 59). In cats, the most frequently reported extraparenchymal tumors of the spinal cord are lymphomas and osteosarcomas (5), of which especially lymphomas might share morphologic similarities with diffuse oligodendrogliomatosis. A possible infectious disease that needs to be ruled out in cats is feline infectious peritonitis (FIP) (60, 61).

Treatment is often difficult due to the lack of specific clinical, radiologic, and laboratory diagnostic criteria, confusion with more frequently occurring infectious, autoimmune or metabolic diseases, the progressive nature with a high mitotic rate, and widespread diffusion, frequently leading to secondary lesions like hydrocephalus (15, 20, 53). However, there are reports of combined radiation- and chemotherapy in humans that improve survival (62, 63). To date, there are no reports of specific PDLG treatment in domestic animals, but long-term remission of an anaplastic oligodendroglioma in a cat was achieved through combined radio- and chemotherapy (64).

## Conclusion

The present report describes the first case of diffuse leptomeningeal oligodendrogliomatosis in a young adult cat including the evaluation of a broad immunohistochemical panel. This rare condition in humans and domestic animals should be considered as a differential diagnosis in animals with neurological symptoms, diffuse leptomeningeal enhancement on MRI and diffuse intrameningeal cell infiltration. Histopathological examination in combination with immunohistochemical staining are required to confirm the diagnosis and further clinical, pathological and molecular data are needed for a better understanding of this disease in domestic animals.

## Data Availability Statement

The original contributions presented in the study are included in the article/Supplementary Material, further inquiries can be directed to the corresponding author/s.

## Ethics Statement

Ethical review and approval was not required for the animal study because the presented case derived from an animal which was submitted for routine diagnostic services in order to determine the cause of disease. Written informed consent was obtained from the owners for the participation of their animals in this study.

## Author Contributions

JW conducted clinical and laboratory examinations and treatment. EC, MH-T, and CP conducted pathological and immunohistochemical examinations and case documentation. EC drafted the manuscript. All authors contributed to manuscript revision, read, and approved the submitted version.

## Funding

This publication was supported by Deutsche Forschungsgemeinschaft and University of Veterinary Medicine Hannover, Foundation within the funding programme Open Access Publishing.

## Conflict of Interest

The authors declare that the research was conducted in the absence of any commercial or financial relationships that could be construed as a potential conflict of interest.

## Publisher's Note

All claims expressed in this article are solely those of the authors and do not necessarily represent those of their affiliated organizations, or those of the publisher, the editors and the reviewers. Any product that may be evaluated in this article, or claim that may be made by its manufacturer, is not guaranteed or endorsed by the publisher.
