# Supplementary material for: Case Report: Primary Diffuse Leptomeningeal Oligodendrogliomatosis in a Young Adult Cat
Source: Front Vet Sci. 2021 Dec 15;8:795126. doi: 10.3389/fvets.2021.795126 (PMC8714914; doi:10.3389/fvets.2021.795126)
Supplement: Supplementary file 1 [file Image_1.pdf]

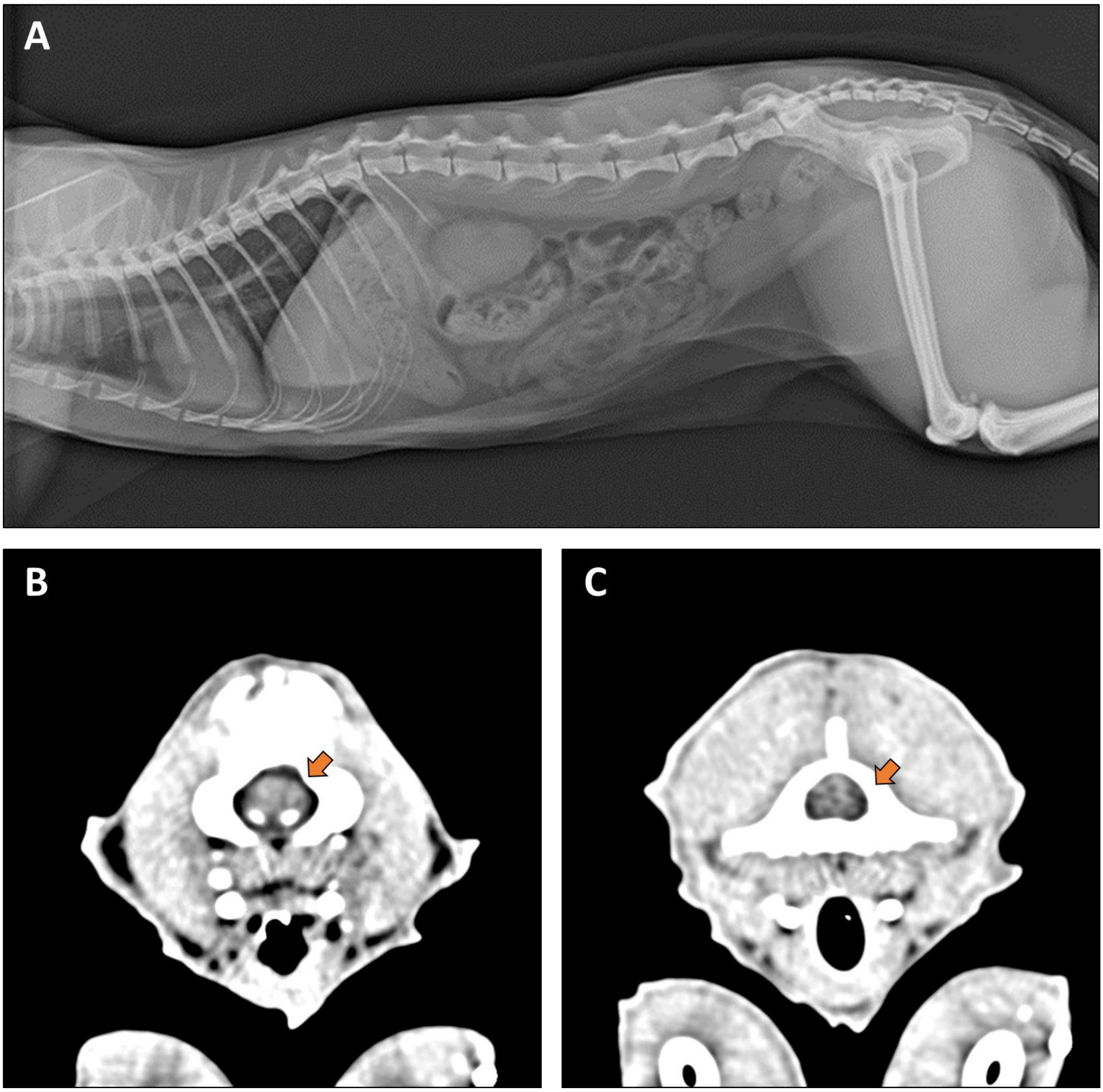

Supplementary Figure 1 | Imaging diagnostics, cat. **(A)** Latero-lateral radiograph of the thoracic cavity and the abdomen lacking abnormalities in the skeleton and the spinal cord. **(B, C)** Transversal computed tomography sections of the cranial cervical spinal cord. No abnormalities were detected in the spinal canal (arrows).
